# Complete Genome Sequence of Klebsiella sp. CTHL.F3a, a Cellulolytic Strain Isolated from Korean Kimchi

**DOI:** 10.1128/mra.00377-22

**Published:** 2022-07-11

**Authors:** C. T. H. Lee, G. K. K. Lai, S. D. J. Griffin, F. C. C. Leung

**Affiliations:** a Shuyuan Molecular Biology Laboratory, The Independent Schools Foundation Academy, Hong Kong SAR, China; Loyola University Chicago

## Abstract

The cellulolytic strain Klebsiella sp. CTHL.F3a was isolated from kimchi (Korean fermented cabbage/vegetables). Its complete genome sequence (6,146,223 bp, GC content of 55.21%), comprising a chromosome and a single plasmid, was established through hybrid assembly.

## ANNOUNCEMENT

Members of the Klebsiella oxytoca species complex (KoSC) may be encountered as human commensals and opportunistic pathogens (1) but are common plant-growth-promoting endophytes (2, 3) that fix nitrogen (4), solubilize soil-bound phosphate (5), and also metabolize cellulose (3, 6). Klebsiella sp. CTHL.F3a, isolated from kimchi, demonstrates significant cellulolytic activity at ambient temperatures and hence may be useful for the pretreatment of lignocellulosic waste for biofuel production (7).

Six samples of fresh kimchi (Korean fermented cabbage/vegetables) from Hong Kong markets were screened for cellulolytic bacteria using carboxymethylcellulose (CMC) agar, as described previously (8). Twelve colonies were further assessed for growth (optical density at 600 nm [OD_600_] via a BMG Labtech CLARIOstar Plus instrument) in Bushnell-Haas medium (BHM) (9) containing either 1% CMC or 1% cellobiose as the sole carbon source. CTHL.F3a, showing the fastest growth on both substrates, was passaged for 7 generations on Luria agar before DNA extraction from overnight culture in Luria broth (all at 27°C) using Invitrogen’s PureLink genomic DNA mini kit, following the manufacturer’s instructions.

Paired-end short-read sequencing libraries, prepared using the NexteraXT DNA library preparation kit, were sequenced via the Illumina MiSeq platform with v3 chemistry (2 × 300 bp). Adapter sequences were removed, and 1,416,362 raw reads were quality filtered and trimmed using Trimmomatic v0.32 (10) to give 704,432 read pairs with an average length of 217 bp (approximately 153 Mbp). Long-read libraries, prepared from the same extracted DNA using a genomic DNA (gDNA) rapid barcoding kit (SQK-RBK004), were sequenced using a SpotON flow cell (vR9) and MinION sequencer, with data acquisition using MinKNOW v3.1.8 software and base calling by Guppy v2.1.3 (all from Oxford Nanopore). The final long-read data set, trimmed by Porechop v0.2.4 (11, 12), totaled 310,076 reads (2.16 Gbp) with a median length of 4,365 bp (*N*_50_, 12,200 bp). Default parameters were used for all software unless otherwise specified.

The Illumina and Nanopore datasets were combined using Unicycler v0.4.3 (13) to yield a circular chromosome (5,995,415 bp, GC content of 55.34%) and a circular plasmid (150,808 bp, GC content of 49.79%) (rotated by default to start with *dnaA*/*repA* on the forward strand), with genome coverage of 81×, which were submitted to NCBI PGAP v5.0 (14) and to PATRIC (15) for annotation.

The CTHL.F3a chromosome has an average nucleotide identity (by OrthoANIu online at https://www.ezbiocloud.net/tools/ani) of 99.60% (16) with rice endophyte Klebsiella sp. BDA134-6 (GenBank accession number CP064784) (17). It contains five copies of beta-glucosidase (EC 3.2.1.21); nine copies of 6-phospho-beta-glucosidase (EC 3.2.1.86); and both type Ib and type IIa *bcs* operons (18), punctuated by type I toxin-antitoxin system LdrD (19). Phyre2 (20) finds the (type 1b) endo-beta-1,4-glucanase (cellulase) (EC 3.2.1.4) structurally similar to the GH8 glycosyl hydrolase Cel10, which was characterized previously in a strain of Klebsiella pneumoniae recovered from the gut of a Chinese bamboo rat (Rhizomys sinensis) (21).

Plasmid pCTHL.F3a was classified as IncFIB(K)_30 (clade II) by the KpVR Web-based tool (https://bioinfo-mml.sjtu.edu.cn/KpVR/index.php) (22). It contains the RelBE and PsiAB toxin-antitoxin systems (23) but no antimicrobial resistance genes.

### Data availability.

Complete genome sequences and raw sequence data for Klebsiella sp. CTHL.F3a are available through NCBI under BioProject PRJNA758781, with GenBank accession numbers CP082360 (chromosome) and CP082361 (plasmid) and SRA accessions SRX12151552 (MiSeq) and SRX12151553 (MinION). Klebsiella sp.
